# Maintaining balance against force perturbations: impaired mechanisms unresponsive to levodopa in Parkinson's disease

**DOI:** 10.1152/jn.00996.2015

**Published:** 2016-04-20

**Authors:** Irene Di Giulio, Rebecca J. St George, Eirini Kalliolia, Amy L. Peters, Patricia Limousin, Brian L. Day

**Affiliations:** ^1^Sobell Department of Motor Neuroscience and Movement Disorders, Institute of Neurology, University College London, London, United Kingdom;; ^2^School of Medicine, University of Tasmania, Hobart, Australia; and; ^3^St. Luke's Hospital, Thessaloniki, Greece

**Keywords:** Parkinson's disease, balance, postural instability, dopaminergic pathways, pull test, levodopa

## Abstract

There is evidence that postural instability associated with Parkinson's disease (PD) is not adequately improved by levodopa, implying involvement of nondopaminergic pathways. However, the mechanisms contributing to postural instability have yet to be fully identified and tested for their levodopa responsiveness. In this report we investigate balance processes that resist external forces to the body when standing. These include in-place responses and the transition to protective stepping. Forward and backward shoulder pulls were delivered using two force-feedback-controlled motors and were randomized for direction, magnitude, and onset. Sixteen patients with PD were tested OFF and ON levodopa, and 16 healthy controls were tested twice. Response behavior was quantified from 3-dimensional ground reaction forces and kinematic measurements of body segments and total body center-of-mass (CoM) motion. In-place responses resisting the pull were significantly smaller in PD as reflected in reduced horizontal anteroposterior ground reaction force and increased CoM displacement. Ankle, knee, and hip moments contributing to this resistance were smaller in PD, with the knee extensor moment to backward pulls being the most affected. The threshold force needed to evoke a step was also smaller for PD in the forward direction. Protective steps evoked by suprathreshold pulls showed deficits in PD in the backward direction, with steps being shorter and more steps being required to arrest the body. Levodopa administration had no significant effect on either in-place or protective stepping deficits. We conclude that processes employed to maintain balance in the face of external forces show impairment in PD consistent with disruption to nondopaminergic systems.

## Abstract (precis)

*We introduce a new method to investigate postural instability in Parkinson's disease (PD) using computer-controlled motors to deliver precise pulls to the shoulders of subjects while standing. It mimics the clinical pull test but uses forces with unpredictable timing, direction, and magnitude. It revealed a number of balance control deficits in PD. Notably, the identified deficits were not significantly altered by levodopa medication, suggesting that disruption to nondopaminergic systems contributes to postural instability in PD*.

## NEW & NOTEWORTHY

*We introduce a new method to investigate postural instability in Parkinson's disease (PD) using computer-controlled motors to deliver precise pulls to the shoulders of subjects while standing. It mimics the clinical pull test but uses forces with unpredictable timing, direction, and magnitude. It revealed a number of balance control deficits in PD. Notably, the identified deficits were not significantly altered by levodopa medication, suggesting that disruption to nondopaminergic systems contributes to postural instability in PD*.

the postural instability associated with Parkinson's disease (PD) can have serious consequences because it is linked with falls (Balash et al. 2005; Burn 2002; Frenklach et al. 2009; Schrag et al. 2015), increased morbidity, and increased mortality (Hely et al. 2005). It poses a difficult challenge for clinicians because postural instability becomes more prevalent with disease progression (Kim et al. 2013; Viitasalo et al. 2002) while at the same time becoming relatively less responsive to levodopa (Koller et al. 1989; Steiger et al. 1996). This has led to the suggestion that disruption of nondopaminergic pathways may play a key role in the evolution of postural instability in PD (Bonnet et al. 1987; Muller et al. 2013; Pahapill and Lozano 2000; Schrag et al. 2015; Viitasalo et al. 2002; Zweig et al. 1989). If true, this would have important implications for therapeutic strategies aimed at alleviating the problem. However, postural instability is a broad phenomenon presumably stemming from different pathophysiological processes. These have yet to be fully identified and quantified, arguably a necessary requirement for critical examination of the nondopaminergic hypothesis. In this study we investigated this hypothesis with respect to the mechanisms that act to preserve balance when an external force perturbs the body.

Maintaining balance in the face of an external force can involve three processes. The first process is responsible for generating forces between the feet and the floor to counteract the perturbing force and decelerate body motion. During the execution of this “in-place” response, a second process assesses the current body state to establish whether the body is falling or is likely to fall in the near future. If this threshold is crossed, then a third process is engaged that is responsible for executing a step with the appropriate metrics to alter the base of support and recapture the falling body. This cycle is then repeated until the body is in equilibrium. To our knowledge, impairments within each of these processes and their dependence on dopaminergic pathways have not been studied under identical conditions in the same sample of patients. Most other studies in this field have been quite diverse with respect to the types of postural perturbations employed and the evoked behavioral responses measured. Perturbations have consisted of platform translations (de Kam et al. 2014; Dimitrova et al. 2004; Horak et al. 1992, 1996; Jacobs and Horak 2006; King et al. 2010; Lee et al. 2013; Smulders et al. 2014), platform rotations (Bloem et al. 1996; Carpenter et al. 2004, Oude Nijhuis et al. 2014; Schieppati and Nardone 1991), or pulls to the body (Foreman et al. 2012; Jöbges et al. 2004; McVey et al. 2013). These perturbations have been employed to elicit postural in-place responses (Bloem et al. 1996; Carpenter et al. 2004; Horak et al. 1992, 1996; Oude Nijhuis et al. 2014; Schieppati and Nardone 1991), step responses (de Kam et al. 2014; Foreman et al. 2012; Jacobs and Horak 2006; Jöbges et al. 2004; King et al. 2010; Lee et al. 2013; McVey et al. 2013; Smulders et al. 2014), or both (Dimitrova et al. 2004). Some of these studies have investigated the effects of levodopa, but these have been distributed between different perturbation types and different behavioral responses (Bloem et al. 1996; Carpenter et al. 2004; de Kam et al. 2014; Foreman et al. 2012; Horak et al. 1992, 1996; Jacobs and Horak 2006; King et al. 2010).

Our aims were to investigate, in a single sample of PD patients, impairments of each of the three balance processes that are used to resist external forces and to establish the levodopa responsiveness of the identified impairments. To achieve this, we employed a laboratory version of the clinical pull test. The clinical pull test, which is used as part of the motor examination within the Unified Parkinson's Disease Rating Score (UPDRS), requires that the patient is pulled backward by the clinician with sufficient force to evoke one or more steps to prevent a fall. However, this clinical test suffers from a number of weaknesses, including uncontrolled force profiles, highly predictable perturbation timing and direction, and coarse scoring of performance. Above all, it only assesses the recovery process once a loss of balance has occurred; it does not address the ability to resist the perturbing force in the first place. Our laboratory version of the pull test circumvents these drawbacks by using computer-controlled motors to deliver precise forward and backward perturbing forces. This allows the delivery of balance-threatening perturbations that are unpredictable in their timing, amplitude, and direction. By measuring whole body kinematic responses and ground reaction forces, we are able to replace the discrete clinical scoring system with continuous measures of performance. By varying the magnitudes of the pull tailored to each individual, we are able to isolate the three processes of external force resistance that are employed to maintain standing balance. These constitute *1*) the first line of defense of generating resistance without moving the feet, i.e., in-place response; *2*) the decision to adopt a stepping strategy, i.e., stepping threshold; and *3*) the execution of steps as a second line of defense to recover balance, i.e., stepping response. We have determined which aspects of these behaviors are impaired in patients with PD by comparing them with age-matched controls. We investigated whether these impairments are dependent on dopaminergic pathways by measuring the quantitative effects of levodopa administration.

## METHODS

### Ethics

Procedures were approved by the University College London Hospitals National Health Service Trust ethics committee. Participants gave written, informed consent to the experiment, which conformed to the Declaration of Helsinki.

### Participants and Clinical Rating

Sixteen patients with PD patients (4 women; age, 66.8 ± 6.5 yr; height, 1.69 ± 0.11 m; weight, 77.9 ± 14.3 kg, means ± SD) were recruited from the National Hospital for Neurology and Neurosurgery. The patients had a mean disease duration of 8.3 ± 6.9 yr, were at Hoehn and Yahr stages 2 to 3, were not implanted with deep brain stimulators, and had not fallen during the 6-mo period prior to testing. Additional patient details are given in Table 1. Sixteen healthy participants (7 women; age, 65.2 ± 8.6 yr; height, 1.70 ± 0.09 m; weight, 74.6 ± 11.8 kg) with no balance or self-reported neurological problems were recruited as controls. Each patient was assessed by an experienced clinician using the part III UPDRS test (motor score). The scoring was undertaken during each experimental session and subsequently checked by the same clinician using video recordings of the test. All participants performed two sessions during a single visit to the laboratory. The PD group was tested first in the OFF state, i.e., after at least 12 h of medication (levodopa) washout, and later in the ON state, i.e., 1 h after taking their equivalent morning dose. Healthy individuals were also tested twice to control for fatigue and/or learning effects. The limitation of this approach is that it assumes any such time-dependent effects are similar for both groups.

**Table 1.** Patient details

						UPDRS III			
Patient Identifier	Age, yr	Disease Duration, yr	Height, m	Weight, kg	Hoen and Yahr Stage	OFF	ON	LED, mg/day	Comorbidity
PTP01	71.1	21	1.67	70	2	28	15	450	None
PTP02	78.2	4	1.7	74.3	3	40	29	650	L knee replacement
PTP03	71.9	5	1.71	102	2	59	38	800	Prostate cancer
PTP04	68.2	6	1.75	78.9	2	20	13	530	None
PTP05	67.7	20	1.55	63.3	2	64	45	850	Lower back pain
PTP06	69.8	20	1.68	62.8	2.5	29	19	1,604	Carotid stenosis, elbow surgery
PTP07	72.1	2	1.54	80.6	2	36	33	150	None
PTP08	74.8	1	1.45	79.8	2	33	24	150	Arthritis, L&R knee replacement, angina
PTP09	62.4	8	1.79	77.9	2	40	26	550	Lower back pain
PTP10	65.8	15	1.64	79.7	2.5	47	27	2,096	Cerebrovascular problems
PTP11	55.6	10	1.78	65.5	2	43	36	1,025	Knee and lower back pain
PTP12	63.4	2	1.76	83	2	40	31	300	Migraine
PTP13	63.9	5	1.61	52	2	40	32	680	None
PTP14	77.8	3	1.74	77	2	34	19	490	None
PTP15	63.6	6	1.86	91.1	2	24	12	750	None
PTP16	58.6	5	1.79	108.5	2	30	24	310	Impulse control disorder

LED, levodopa equivalent dose (Tomlinson et al. 2010).

Only PTP02 used assistive devices for walking.

### Experimental Procedure

**Fig. 1.**
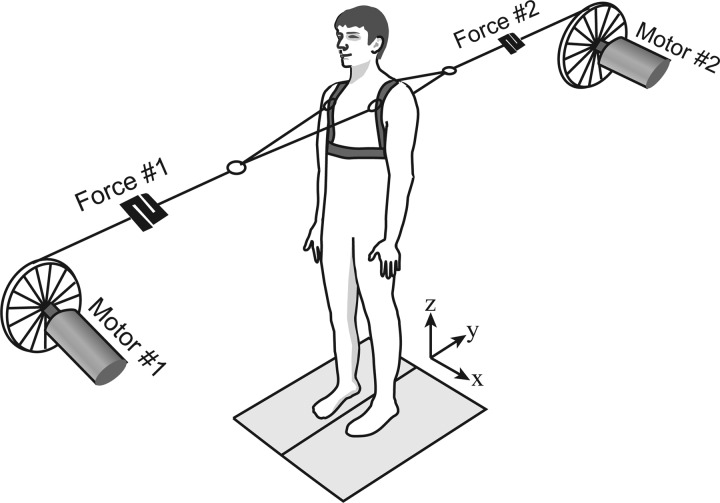
Apparatus. Participants stood on two force platforms and wore a safety harness. Two motors delivered 1-s-long pulls of different force and direction (backward and forward) via kite strings attached at shoulder level. Participants were asked to resist the pull unless one or more steps became necessary to maintain balance.

Participants stood symmetrically in a comfortable position with their feet on separate force plates. The feet positions were marked with chalk to ensure repeatability. Light, inextensible kite strings (Climax Protec 50daN; Ockert, Puchheim, Germany) were connected bilaterally to the anterior and posterior shoulder straps of a full body harness worn by the participant. The harness was also connected to an overhead zip wire, which could arrest a fall but allowed forward and backward steps. A randomized series of anterior and posterior pulls of variable force were delivered via the strings by two motors (Fig. 1), one positioned in front and the other behind at a distance of ∼2.7 m from the participant. In contrast to the clinical pull test, forward and backward pulls were intermixed to minimize expectation of pull direction and to investigate further the reported greater instability in the backward direction in PD (Carpenter et al. 2004; Lee et al. 2013).

The participant's center of pressure (CoP) was calculated and monitored in real time using the eight vertical force signals from the two force plates (4 from each plate under each foot). Before each perturbation trial, real-time CoP was used to ensure the participant was in a stable state as judged qualitatively by CoP oscillations not being excessively high. It was also used to check that their weight distribution was not overly deviated from their preferred position. Their preferred position was measured at the beginning of each session while they stood for 30 s in a comfortable position with a small tensioning force (2 N) applied to the pulling strings (zero net force). The perturbation trial was not initiated and the participant's position or posture was corrected if their CoP had deviated from this preferred position by more than 30 mm forward, 25 mm backward, or 50% of the distance laterally to either foot.

At a random time (2–5 s) before trial onset, the small bidirectional tensioning force (2 N) was applied to the strings. The participants were warned before this occurred and were told that a variable-sized pull would subsequently be delivered in an unpredictable direction a short time later. Participants were instructed to resist the pull without moving their feet unless one or more steps became necessary to avoid a fall, in which case they were asked to try to become stationary as soon as balance was regained. Each trial lasted 7 s and consisted of 2-s pre-pull, 1-s pull (0.1-s ramp to plateau for 0.9-s duration), and 4-s post-pull.

Each session was divided into seven blocks of eight trials each. After each block, the participant was allowed to move about or sit down. For the first block, four fixed pulls of 10 and 20 N in each direction were applied twice in random order to probe the participant's stability. For each subsequent block, four other pulls of variable magnitude (range from 10.5 to 60 N across all participants and conditions), which often induced steps, were randomly intermixed with the four fixed pulls. The fixed pulls of 10 and 20 N were used to assess the participant's in-place responses, whereas the variable magnitude pulls were used to assess stepping threshold as well as stepping response behavior.

#### Threshold determination.

To minimize the test duration, a method was devised that attempted to converge on a participant's stepping threshold, i.e., the pull force that evoked a stepping response 50% of the time, within 12–16 trials per direction. A hybrid method was used that combined a human estimator with a machine algorithm. The human estimator, who was experienced in observing PD patients responding to pulls, provided the initial “best guess” at the pull level that was likely to be above, but close to, a participant's threshold without subjecting him or her to excessive force. Once steps had been evoked, the machine algorithm was used to help and converge on the threshold. After each trial, the operator inserted the outcome (Step or No Step) in custom-built software (LabVIEW; National Instruments, Austin, Texas) that estimated the stepping threshold and selected future pull forces to refine the estimate. However, the operator always reserved the right to intervene and set the forces manually at the beginning of each block if the estimate was deemed to require fine-tuning. If the threshold estimation was not very accurate (SD >1 N) at the end of the 56 pulls, or if the outcome was unclear for a particular pull level, extra targeted pulls (usually no more than 4 in total) were added to resolve the uncertainty.

### Apparatus and Measurements

The pulls were generated by two servomotors (AKM52H-ANCNAA00; Kollmorgen, Radford, VA) running in torque mode and controlled through custom-built LabVIEW software. The motors were tuned to deliver a demanded torque regardless of the participants' movements. Two custom-built strain-gauge force transducers (supported from strings hanging from the ceiling) were placed in series with the kite strings and used to measure the horizontal force administered during each pull (Fig. 1). The three components of ground reaction force and the point of application of the net force vector under each foot was measured using two force plates (models 9281B and 9287; Kistler Instrumente, Winterthur, Switzerland). Forces were sampled at 1 kHz and later downsampled to 100 Hz.

Whole body kinematics were recorded in three dimensions using an active infrared motion capture system (CODA CX1; Charnwood Dynamics, Rothley, UK). Clusters of four infrared-emitting diodes (IRED) were placed on the following segments: feet, shanks, thighs, pelvis, upper back, head, lower arms, and upper arms. A pointer was used to associate the clusters with the following anatomical landmarks: 5th and 1st metatarsal heads, 2nd toe tip, heels, lateral and medial malleoli, lateral and medial femoral epicondyles, greater trochanters, lateral upper edges of the pelvis, navel, clavicle notch, acromions, anterior and posterior aspects of the shoulders, ulnar and radial elbows, ulnar and radial wrists, 12th thoracic vertebra, 7th cervical vertebra, ear meatus, lateral orbital edges of the zygomatic bone, and top of the head. Each cluster's position was marked with washable pen, and landmarks were marked with adhesive stickers to control for cluster movement and artifacts between blocks and sessions. If any cluster was inadvertently moved, the landmarks relative to the cluster were repointed. Kinematic data were sampled at 100 Hz.

### Data Analysis

The motion capture files were imported into Visual 3D (C-Motion; Germantown, MD), and a whole body model was built. The marker trajectories were filtered with a Butterworth zero-lag, low-pass, 12-Hz filter. The segments comprising feet, shanks, thighs, pelvis, torso, head, lower arms, and upper arms were defined for the model. The participant's height and weight were specified to calculate whole body center of mass (CoM). The CoM of the model was obtained from the calculated center of mass of the kinetic segments resolved in the laboratory coordinate system. The center of mass of each segment was calculated using the Demter and Hanavan's model, with each limb segment modeled as a conical frustrum, the pelvis and thorax modeled as elliptical cylinders, and the head modeled as an ellipsoid. Ankle, knee, and hip internal moments were calculated, and these data together with the kinematic model calculations, including landmark locations and joint angles calculated between segments, were exported into Matlab (The MathWorks, Natick, MA).

Trials were divided into Step and No-Step trials offline. The criteria for a Step trial were that *1*) the vertical ground reaction force signal from one force platform dropped below 5 N, and *2*) the corresponding foot moved in the direction of the pull. This defined the initial foot-lift event. Foot landing was identified using a hybrid method. If the foot landed on the force platform, landing time was the frame after foot lift in which the vertical signal first rose above 5 N. If foot events occurred off the force plate, the following kinematic method was used (adapted from Pijnappels et al. 2001). A rigid foot segment was built from landmarks comprising 2nd toe tip, 1st and 5th metatarsal head, heel, and medial and lateral malleoli. Foot landing was defined as the frame after foot lift (>5 frames) when the foot segment anteroposterior (AP) velocity dropped below the mean plus 1.5 times the SD of the pre-pull value for more than 3 frames. When the force and kinematic methods were both available, they yielded a maximum discrepancy of ±2 frames.

Further steps were identified with the extra constraint that the stepping foot had to move past the stance foot in the direction of the pull to exclude foot movements that were not directly involved in arresting body motion. For these steps, foot lift was defined as the first frame in which a foot segment's AP velocity and AP position both exceeded the mean values plus 1.5 times the SD of the previous stance phase for more than 3 frames.

#### Initial posture.

Mean lower, upper, and overall body configurations were measured between 1 and 0.5 s before pull onset (after the strings were tensioned). Although participants were in a state of readiness for the upcoming pull, they did not know its size or direction. Ankle joint, hip joint, and sagittal plane trunk segment angles were calculated from orientations in space of appropriate IRED clusters. As a global measure, the position of the CoP was measured relative to the mean ankle location (midpoint between the left and right ankle joint centers).

#### In-place responses.

Pull forces of 10 and 20 N were used to measure in-place responses, but trials were excluded if a step occurred. Resisting force was calculated as the mean anteroposterior (AP) horizontal ground reaction force (summed from the 2 force plates) measured between pull onset and 0.5 s later. CoM displacement was measured as displacement in the AP direction at the end of the 1-s pull relative to its mean pre-pull position (from −150 to −50 ms), normalized by the participant's body mass. For each joint, the left and right moments in the sagittal plane were averaged after subtraction of the pre-pull baseline to remove any offset. The mean value between pull onset and 0.5 s later was calculated and normalized by the pull force to allow the responses to the two pull levels to be combined.

#### Stepping threshold.

Stepping threshold was estimated from all the trials and defined as the pull force that induced a step 50% of the time. This quantity was calculated by first fitting a sigmoid curve to the pull force vs. step outcome graph (see Fig. 4*A*), differentiating the curve, and measuring the force at which its peak occurred. This value was then normalized by dividing by the participant's body mass. The rationale for this normalization was based on the results of a stepwise multilinear regression analysis in which sex, age, height, body mass, and height × body mass were included as potential predictors of threshold. The results suggested that body mass is the main factor consistently influencing threshold.

#### Stepping responses.

Foot-lift latency was the time relative to pull onset when the vertical ground reaction force signal dropped below 5 N. Momentum at foot lift was determined as the AP velocity of the CoM at foot-lift onset multiplied by the participant's body mass. Step length was defined as the foot displacement with respect to the hip and was measured as the difference in the AP position of the foot's leading edge (2nd toe tip for forward pulls and heel for backward pulls) between first foot-lift and first foot-landing times minus the difference in the hip AP position at the same time points. Number of steps was limited to three, because an operator often intervened when more steps occurred.

### Statistical Analysis

Statistical analysis was performed using Statistica (Dell Statistica 12; Tulsa, OK). A Wilcoxon test was used to assess the difference between OFF- and ON levodopa for the UPDRS scores. A general linear model repeated measure (GLMRM) design was used to analyze the other quantities, but with data for forward and backward pulls being analyzed separately. This separation of direction was because the pull forces for evoking steps were higher in the forward than the backward direction [direction: *F*(1,30) = 10.72, *P* = 0.003]. For in-place responses, pull size (10 and 20 N) and session (*1* and *2*) were fixed factors, and group (PD and healthy controls) was the between-subjects factor in the GLMRM model. For stepping threshold, stepping responses, and initial posture, the factors included in the model were session and group. Significant interactions were explored further using Tukey's honestly significant difference post hoc test. Pearson's correlation coefficient was used to investigate correlations between different impairments and between impairments and disease duration. Spearman's rank correlation was used to investigate the relationship between the number of steps induced by backward perturbations and the UPDRS clinical pull test item score. Alpha was set at 0.05 for significance. Unless otherwise stated, results are means ± SE.

## RESULTS

The mean part III UPDRS score significantly improved with levodopa, from 37.9 (±11.7 SD) when OFF to 26.4 (±9.4 SD) when ON (*P* < 0.001).

### Initial Posture

We were unable to detect significant group differences or effects of medication on initial body posture measured during the period immediately preceding each pull. Four independent variables were measured: *1*) anterior position of CoP relative to ankle joint center [PD vs. control: 61.5 ± 3.2 vs. 56.2 ± 2.5 mm; *F*(1,30) = 0.89, *P* = 0.354], *2*) ankle flexion angle [72.3 ± 0.8 vs. 70.7 ± 0.7 deg, *F*(1,30) = 2.08, *P* = 0.160], *3*) hip flexion angle [12.3 ± 3.9 vs. −0.11 ± 2.6 deg, *F*(1,30) = 3.61, *P* = 0.067], and *4*) torso sagittal plane angle [19.6 ± 0.2 vs. 19.4 ± 0.2 deg, *F*(1,30) = 0.43, *P* = 0.515]. These quantities did not change significantly with levodopa.

### In-Place Responses

**Fig. 2.**
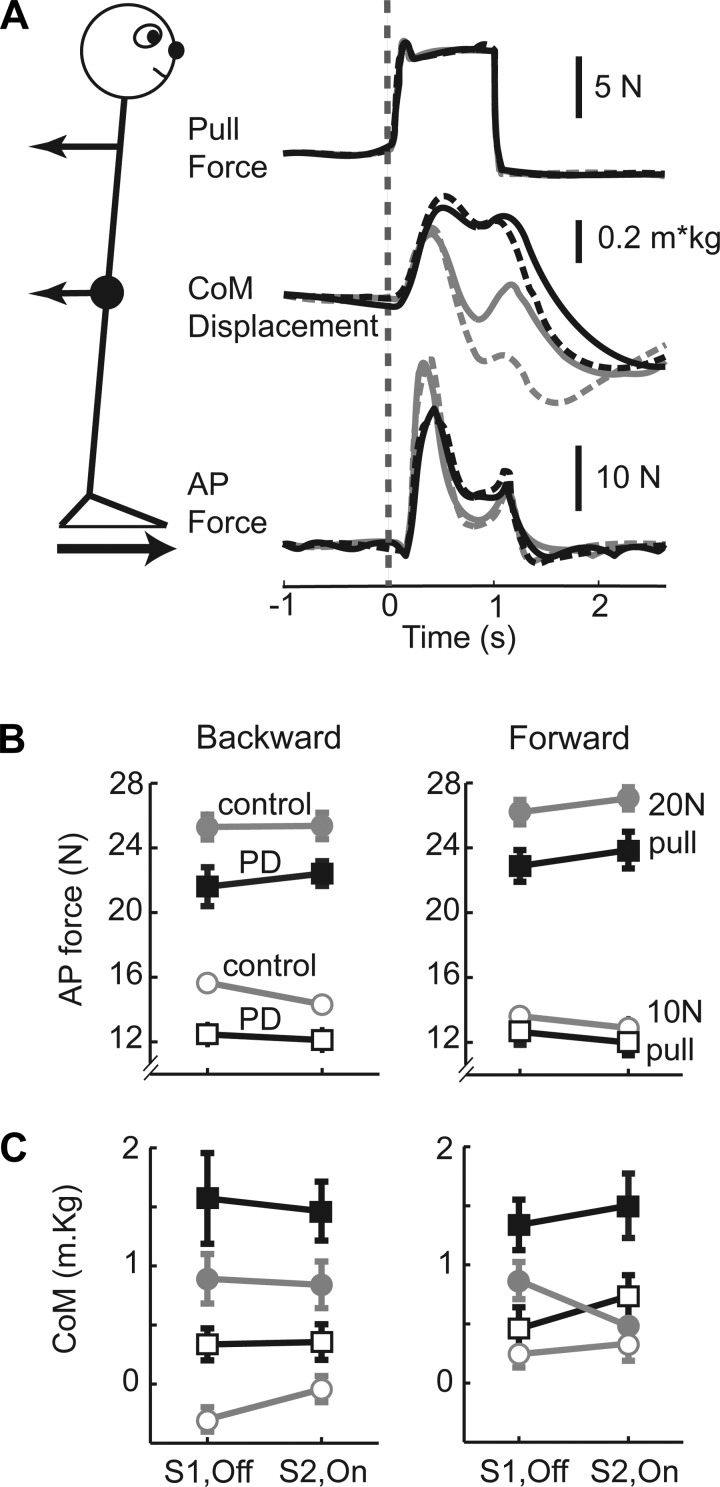
Whole body in-place responses. *A*: group mean (*n* = 16) responses for PD (black lines) and controls (gray lines) to 10-N pulls in the backward direction for *session 1* (PD OFF; dashed lines) and *session 2* (PD ON; solid lines). *Top*, perturbation force; *middle*, center of mass (CoM) displacement normalized by body mass; *bottom*, anteroposterior (AP) ground reaction force. *B*: group mean (±SE) AP reaction force measured between 0 and 500 ms after pull onset for PD (squares, black lines) and controls (circles, gray lines). Data from force levels of 10 N (open symbols) and 20 N (filled symbols) are shown separately for each session (S1, OFF; S2, ON) and each direction (backward, forward). *C*: same conventions as *B* for the mean normalized CoM displacement measured at the end of perturbation.

Figure 2*A* shows each group's mean in-place responses for backward 10-N pulls, which typified the general behavior. Shortly after pull onset, the body initially swayed in the direction of the pull and then was arrested or reversed while the pull force was maintained at a constant level. This was achieved by the generation of an opposing horizontal ground reaction force that was initiated after a latent period of around 150 ms. In general, PD patients and control participants behaved similarly except that patients exerted a smaller resisting force and consequently swayed further. This behavior in response to two force levels over two sessions is quantified and analyzed in detail in the following sections. However, it should be noted that with 20-N pulls in *session 1*, but not *session 2*, one patient (PTP05) always stepped in the backward direction, whereas another (PTP13) always stepped in the forward direction. This resulted in missing data for the resisting force and CoM displacement measurements, which automatically excluded these patients' contribution to the overall repeated-measures analysis in one direction each.

#### Resisting force.

On average, PD patients exerted a lower resisting force (measured over the initial 0.5 s of pull; Fig. 2*B*) compared with controls [group: backward, *F*(1,29) = 17.15, *P* < 0.001; forward, *F*(1,29) = 5.15, *P* = 0.031]. The resisting force was lower for 10- compared with 20-N pulls [size: backward, *F*(1,29) = 365.95, *P* < 0.001; forward, *F*(1,29) = 643.94, *P* < 0.001]. There was not a significant main effect of session for either direction.

Some significant interactions were present. The two groups responded differently to the two sizes of pull, but only in the forward pull direction [group × size: forward, *F*(1,29) = 5.50, *P* = 0.026]. Post hoc comparisons showed that this stemmed from there being a significant difference between PD and controls for the 20-N pulls (*P* = 0.017) that was larger compared with the 10-N pulls, with the latter failing to produce a significant difference between groups (*P* = 0.812). In both directions, there was an interaction between the size of the pull and the session [size × session: backward, *F*(1,29) = 9.59, *P* = 0.004; forward, *F*(1,29) = 11.37, *P* = 0.002], indicating that for 20-N pulls, a higher force was produced in *session 2* compared with *session 1*, and vice versa for 10-N pulls.

#### CoM displacement.

Body sway produced by the pull was measured from changes in CoM position (Fig. 2*C*). The main effects mirrored those of the force response. Patients swayed more than controls for both directions of pull [group: backward, *F*(1,29) = 8.58, *P* = 0.007; forward, *F*(1,29) = 5.71, *P* = 0.024]. Both groups swayed more for 20- than for 10-N pulls [size: backward, *F*(1,29) = 44.76, *P* < 0.001; forward, *F*(1,29) = 33.86, *P* < 0.001]. There was not a significant main effect of session for either direction.

The interactions were in line with those of the force response. Thus the difference between the two groups depended on the pull size, but only in the forward direction, with the difference being greater at the higher pull force [group × size: forward, *F*(1,29) = 4.49, *P* = 0.043]. Also, in the forward direction (with a trend in the backward direction) there was greater overall increase in CoM sway in *session 2* relative to *session 1* for 10-N pulls compared with 20-N pulls [size × session: backward *F*(1,29) = 3.92, *P* = 0.057; forward, *F*(1,29) = 8.91, *P* = 0.006].

#### Joint moments.

**Fig. 3.**
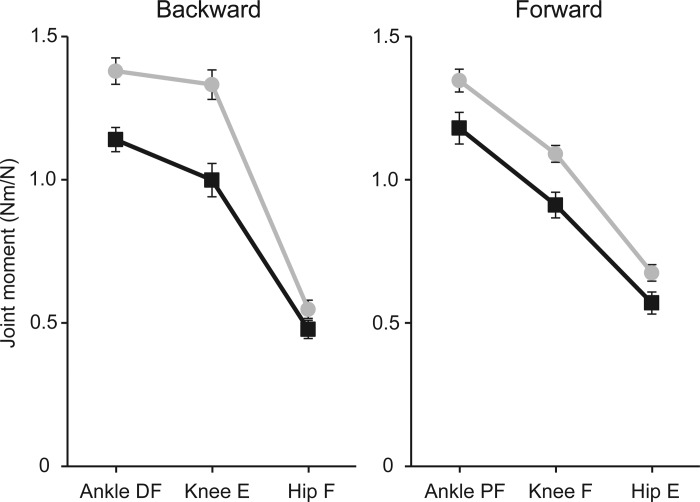
In-place joint moments. Combined left and right joint moments in the sagittal plane were measured between 0 and 500 ms after pull onset. Moments were normalized by pull force and averaged across 10- and 20-N pulls and across sessions. Group means (±SE) are shown separately for backward (*left*) and forward (*right*) perturbations in PD (squares, black lines) and controls (circles, gray lines). Direction of moments is shown as dorsiflexion (DF) or plantarflexion (PF) for ankle and extension (E) or flexion (F) for knee and hip.

Ankle, knee, and hip moments, normalized by pull force and combined for the two pull levels, are shown in Fig. 3. These data have also been collapsed across the two sessions because there were no significant effects of session for either direction. The moments varied with joint type in both directions [joint: backward, *F*(2,60) = 350.43, *P* < 0.001; forward, *F*(2,60) = 564.40, *P* < 0.001], and the PD group produced smaller joint moments than controls in both directions [group: backward, *F*(1,30) = 18.248, *P* < 0.001; forward, *F*(1,30) = 7.98, *P* = 0.008]. For backward pulls only, there was an interaction between group and joint indicating that the difference between the two groups varied according to the joint [group × joint: backward, *F*(2,60) = 7.22, *P* = 0.002]. The largest difference was observed for the knee extension moment, which in PD was 75% of control values.

### Stepping Threshold

**Fig. 4.**
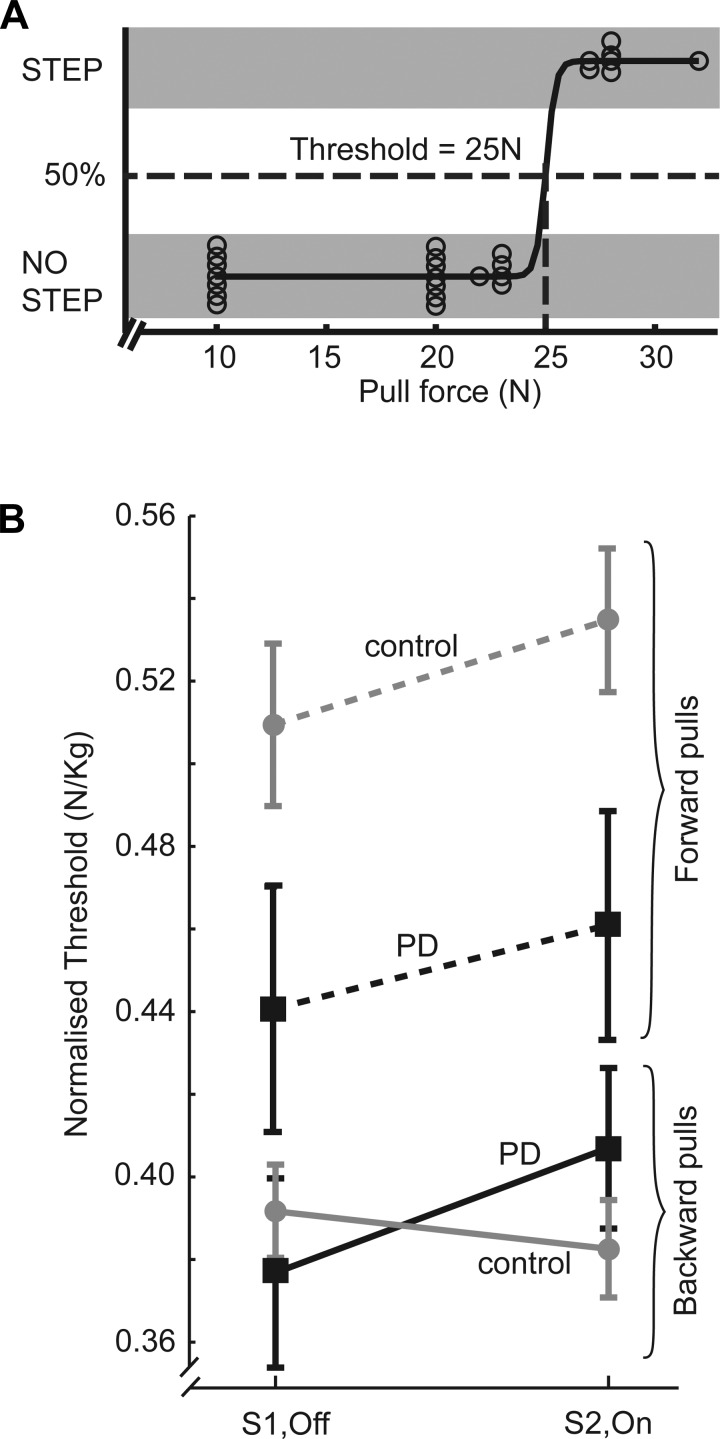
Stepping threshold. *A*: example of sigmoid curve using all trials collected in one session from a representative participant for calculation of stepping threshold (before normalization). *B*: group mean (±SE) stepping threshold normalized by body mass in each session (S1, OFF; S2, ON) for PD (black squares) and controls (gray circles) shown separately for backward (solid lines) and forward (dashed lines) pulls.

In the forward direction (Fig. 4*B*), PD patients' stepping threshold was consistently lower than that of controls [group: forward, *F*(1,30) = 4.97, *P* = 0.033]. In the backward direction (Fig. 4*B*), the only effect was an interaction [group × session: backward, *F*(1,30) = 5.90, *P* = 0.021]. This reflected a relative increase in threshold on the second session for PD patients compared with controls and hints at an effect of levodopa. However, post hoc comparisons showed that PD patients did not differ from controls in *session 1* (*P* = 0.926) or in *session 2* (*P* = 0.747), and PD threshold did not change significantly with levodopa (*P* = 0.060).

### Stepping Responses

**Fig. 5.**
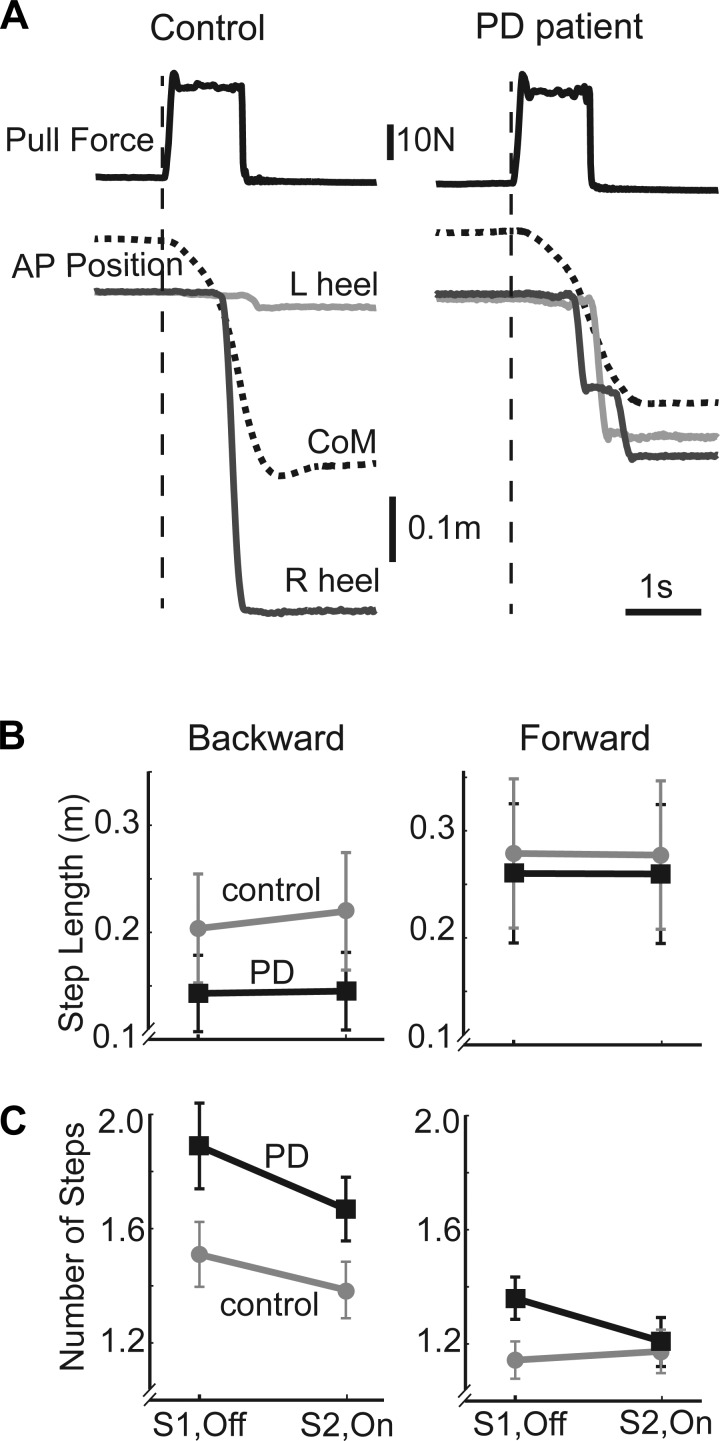
Stepping responses. *A*: representative PD and control participants for 29-N backward pull. *Top*, perturbation force (solid black lines); *bottom*, AP position of CoM (dashed black), and heels (R, right, solid dark gray lines; L, left, solid light gray lines). Although the pulls were identical and the body displacement (CoM trajectory) and foot-lift time were similar, the PD patient took multiple short steps, whereas the control participant took a single longer step. *B*: group mean (±SE) length of first step for PD patients (squares, black lines) and controls (circles, gray lines) for each session (S1, OFF; S2, ON) in the backward (*left*) and forward (*right*) directions. *C*: same conventions as *B* for group mean (±SE) number of steps taken per trial.

Figure 5*A* illustrates contrasting stepping behavior of a representative PD patient and a control participant to a suprathreshold backward pull. These single trials were chosen because they had the same level and direction of pull and produced very similar initial CoM trajectories and latencies of first foot lift. However, stepping execution differed because the PD participant took smaller and more steps than the control.

#### Step length.

Mean PD step length was smaller than that of controls (Fig. 5*B*), but only in the backward direction [group: backward, *F*(1,30) = 6.07, *P* = 0.020]. There was neither a main effect of session nor an interaction, indicating a lack of effect of levodopa on step length.

#### Number of steps.

In the backward direction (Fig. 5*C*), PD patients took more steps than controls [group: backward, *F*(1,30) = 4.28, *P* = 0.047], whereas fewer steps were taken in *session 2* than *session 1* for both groups [session: backward, *F*(1,30) = 8.99, *P* = 0.005]. In the forward direction (Fig. 5*C*), there was only an interaction present, suggesting a possible effect of levodopa on number of steps taken [group × session: forward, *F*(1,30) = 4.89, *P* = 0.035]. However, post hoc comparisons showed no difference between PD OFF compared with controls in *session 1* (*P* = 0.197) and no significant effect of levodopa in PD (*P* = 0.064).

#### Initial conditions.

Differences in step execution between groups could not readily be explained by differences in pull forces or in behavior at the point of step initiation. Stepping-trial pull force did not differ between groups (PD vs. control: backward: *session 1*, 29.4 ± 2.0 vs. 29.8 ± 1.8 N; *session 2*, 31.4 ± 2.3 vs. 32.4 ± 1.2 N; forward: *session 1*, 34.0 ± 2.5 vs. 37.2 ± 1.5 N; *session 2*, 37.2 ± 2.3 vs. 39.5 ± 1.8 N), although the pull forces were higher in *session 2* [session: *F*(1,30) = 15.90, *P* < 0.001]. There were no significant group differences for foot-lift latency (PD vs. control: backward, 0.96 ± 0.04 vs. 0.93 ± 0.04 s; forward, 1.03 ± 0.03 vs. 1.12 ± 0.05 s) or body momentum at foot lift (PD vs. control: backward, 13.6 ± 0.9 vs. 13.9 ± 0.9 kg·m/s; forward, 21.5 ± 1.4 vs. 24.3 ± 1.5 kg·m/s). There were no effects of session for these measures.

### Correlations

In the PD group there was a negative correlation between step length and number of steps (OFF, *R* = −0.626, *P* = 0.009; ON, *R* = −0.587, *P* = 0.017) in the backward direction, suggesting that the small step length was functionally inadequate. In the OFF or ON state, the severity of the two main PD deficits, i.e., decreased in-place resisting force and decreased step length, did not correlate with each other in either direction. Disease duration did not correlate with the severity of either deficit. The number of steps induced by backward perturbations correlated with the clinical pull test UPDRS score when ON (Spearman's ρ = 0.721, *P* = 0.002), but not when OFF (Spearman's ρ =−0.025, *P* = 0.926).

## DISCUSSION

In this study we measured balance responses to external forces applied to the torso at the level of the shoulders. To our knowledge, controlled shoulder perturbations delivered by motors have not previously been used in this way to study PD balance deficits, even though manual pulls to the shoulders are routinely used in clinical practice as a test component of the UPDRS. We believe, also, that this study is the first to analyze in the same PD patients the impairments of both in-place and stepping responses, together with the levodopa responsiveness of those impairments, using one perturbation method. The majority of previous laboratory-based studies of PD balance control have employed platform perturbations that either translate the feet or rotate the ankles to destabilize the body, some of which have measured in-place responses and others stepping responses (see Introduction for references). Although we refer to the results of these studies when they are relevant to the discussion, a direct comparison between responses to platform perturbations and shoulder pulls has limitations because of the different sensory inputs evoked by the two types of stimuli. The different sites of force application give rise to different cutaneous and proprioceptive patterns of input, while perturbations at the level of the floor compared with shoulder generally will lead to different head-in-space trajectories, and hence different vestibular and visual inputs.

The results of the current study revealed differences between PD patients and healthy participants for both the in-place and stepping responses used to maintain balance. This was despite the limitation that we studied patients who were only moderately affected by the disease (Hoehn and Yahr stage 2–3). Before discussing the possible involvement of dopaminergic pathways, we first address the question as to whether these differences represent true impairments of the underlying mechanisms.

### Is the In-Place Balance Mechanism Impaired in PD?

When relatively small forces were used to perturb the body (10 and 20 N), the PD in-place responses were smaller than control responses, leading to increased displacements of the body in the direction of the pull, consistent with results from some platform perturbation studies (Bloem et al. 1996; Carpenter et al. 2004; Dimitrova et al. 2004; Oude Nijhuis et al. 2014). However, we cannot immediately conclude that the reduced PD response was caused by an impaired mechanism. Most PD patients were able to maintain sufficient resistive force throughout these perturbations to prevent a step. Arguably, therefore, their response was adequate for this specific task. On the other hand, two patients always stepped with 20-N pulls (one in the backward and the other in the forward direction), suggesting that their in-place responses were functionally inadequate. A similar deficit became apparent for the PD group as a whole when we increased the pull force to determine the threshold level required to evoke a step. These data showed that PD patients had a lower threshold for steps when being pulled forward, thus favoring an impairment of the in-place balance mechanism.

Against this interpretation was the apparent lack of difference between PD and control thresholds for backward pulls, even though PD in-place responses were reduced in this direction also. The reason behind the threshold discrepancy in the two directions is unclear. One possibility is that the muscles that resist forward pulls (ankle plantarflexors and hip extensors) are relatively weaker than the muscles that resist backward pulls (ankle dorsiflexors and hip flexors). However, this was not apparent in the joint moments measured during 10- and 20-N pulls. If anything, the moments were larger and closer to control values for the forward pulls (see Fig. 3). This is consistent with the finding that although PD ankle muscles are weaker than those of healthy subjects, plantarflexors are relatively less affected than dorsiflexors during eccentric contractions (Pang and Mak 2012). Another possible explanation may be found in the process that drives the decision to take a step when being perturbed by external forces. It has been shown that this decision is not determined simply by the body's mechanical state induced by the external force; contextual factors also play a role. These can exert powerful modulatory effects and lead to steps being taken when they are not strictly necessary (Pai et al. 1998) or delay their onset latency when they are (Rogers et al. 2003). In the current study there may have been greater pressure for the PD patients not to take a step specifically when pulled backward, since step execution in that direction is deficient (see below). Awareness of this deficiency could have elevated the PD backward-stepping threshold compared with their forward threshold.

Overall, the abnormally low in-place responses in both directions together with abnormally low stepping threshold, albeit in one direction, point to impairment of the balance mechanism due to the pathophysiology of PD. The impairment did not appear to be related to response saturation. Thus, when perturbed with 20-N pulls, PD patients produced responses almost twice as large as with 10-N pulls, and larger than the controls' 10-N responses. In other words, PD patients were able to generate the normal resistive force magnitude but for some reason failed to do so. This could mean that the gain of the balance response was set abnormally low in PD. This is an attractive idea that has been postulated to explain other PD motor problems (DeLong 1990; Filion et al. 1988; Konczak et al. 2009). If the gain of the response were lower than normal, one would expect the relationship between response output and stimulus input to have a smaller slope for PD compared with controls. Although caution is required because only two points within the total input-output curve have been measured, our data shown in Fig. 2 support this idea for pulls in the forward direction (reflected in the group × size interaction), but not in the backward direction.

In the backward direction, the slopes, hence gains, appear similar for PD patients and controls. The PD curves give the appearance of being offset below the control curves, as if a fixed part of the input signal had not been processed. This could occur if there was excessive noise somewhere within the sensorimotor loops engaged by the task. In this case the signals would have to exceed the noise band before they could be detected and a response issued. Thus an elevated noise band would require a larger input to produce a given output, leading to the apparent offset input-output relationship without a change in slope seen here. This suggestion is consistent with reports of elevated noise levels caused by loss of selective firing of globus pallidus neurons in animal models of PD (Filion et al. 1988; Konczak et al. 2009).

### Is the Balance-Recovery Stepping Mechanism Impaired in PD?

When higher (suprathreshold) force levels were applied, balance could no longer be maintained by in-place responses and so balance-recovery steps had to be executed as a second line of defense. The balance-recovery steps when measured as foot displacement relative to hip were abnormally small in PD patients, but this was evident only in the backward direction. The same result was obtained when absolute foot displacements were measured (data not shown), although others have failed to find abnormally small steps to a backward perturbation (de Kam et al. 2014; Lee et al. 2013; McVey et al. 2013; Smulders et al. 2014). There was an inverse correlation between step length and number of steps backward, indicating that the step was functionally inadequate because it was less successful in stopping the body. This suggests that the mechanism driving the initial backward step is indeed impaired in PD.

We were unable to find a similar impairment for stepping in the forward direction, in contrast to results from platform translation studies (de Kam et al. 2014; Jacobs and Horak 2006; King et al. 2010; Lee et al. 2013). Does this reflect a true directional difference, or might it be an experimental artifact? One area of concern is that different force levels were employed to evoke steps in the two directions, with that of forward pulls being greater on average than backward pulls. There was no consistent difference in pull magnitude between groups, but the stepping threshold in the forward direction was lower for PD patients than controls. However, this would mean that the forward pull levels were relatively more destabilizing for PD patients. If the PD group had an underlying impairment for stepping in the forward direction, one might expect this bias to exacerbate rather than diminish step execution deficits. This leads us to favor the interpretation that there are true directional differences in PD stepping responses, with recovery steps in the backward direction being more impaired than those in the forward direction.

### Are PD Balance Impairments Dopamine Dependent?

Although the balance mechanisms responsible for the in-place response and the stepping response both showed impairments in PD patients, we were unable to find a correlation between the severities of their respective deficits. This may indicate that the two balance mechanisms to some extent utilize separate neural structures. To investigate the influence of dopaminergic pathways on these mechanisms, we measured the extent to which the two deficits were reduced by administration of levodopa. To establish an effect of levodopa, there had to be a significant improvement in PD performance between *session 2* when ON and *session 1* when OFF, and the improvement had to be significantly greater than that of the control group across their two sessions. For both balance mechanisms we were unable to find a convincing improvement of performance through levodopa administration.

This lack of an effect of levodopa on balance deficits is consistent with previous studies (Bejjani et al. 2000; Bloem et al. 1998; Carpenter et al. 2004; de Kam et al. 2014; Foreman et al. 2012; Guehl et al. 2006; Horak et al. 1996; King et al. 2010; Jacobs and Horak 2006; Rocchi et al. 2002) and could be taken to mean that the deficits are caused by nondopaminergic mechanisms. However, because of the observed trends for increase in backward step threshold and decrease in number of forward steps with levodopa, we cannot rule out the possibility that dopaminergic mechanisms also are involved. If multiple lesions were responsible for the balance impairments, treating one lesion would not necessarily restore function. Therefore, we can reasonably conclude that nondopaminergic lesions play an important role in PD balance impairments (Bohnen et al. 2009; Muller et al. 2013), but dopaminergic lesions may contribute to the process. This would be consistent with the progressive worsening of balance problems and the emergence of nondopaminergic lesions as PD evolves (Bonnet et al. 1987). Cell loss in the pedunculopontine nucleus (PPN) has been shown in PD (Hirsch et al. 1987; Rinne et al. 2008), as well as in other diseases that cause postural abnormalities, such as progressive supranuclear palsy (Bohnen et al. 2009; Zweigh et al. 1989) and multiple system atrophy (Benarroch 2013). Lesions of the PPN elicit PD-like symptoms (Pahapill and Lozano 2000), and a relationship between loss of cholinergic neurons in PPN and severity of PD symptoms has been reported (Zweig et al. 1989). Thus cholinergic neurons in the PPN that are involved in PD progression are good candidates for a role in the balance impairments pinpointed in this study.

## GRANTS

This work was supported by Medical Research Council Grant MR/J013234/1.

## DISCLOSURES

No conflicts of interest, financial or otherwise, are declared by the authors.

## AUTHOR CONTRIBUTIONS

I.D.G., R.J.S.G., P.L., and B.L.D. conception and design of research; I.D.G., R.J.S.G., E.K., and A.L.P. performed experiments; I.D.G. and B.L.D. analyzed data; I.D.G., R.J.S.G., and B.L.D. interpreted results of experiments; I.D.G., R.J.S.G., and B.L.D. prepared figures; I.D.G. and B.L.D. drafted manuscript; I.D.G., R.J.S.G., E.K., A.L.P., P.L., and B.L.D. edited and revised manuscript; I.D.G., R.J.S.G., E.K., A.L.P., P.L., and B.L.D. approved final version of manuscript.
